# Hyaluronidase: structure, mechanism of action, diseases and therapeutic targets

**DOI:** 10.1186/s43556-025-00299-y

**Published:** 2025-07-12

**Authors:** Jiamin Lu, Zheng Zhao, Lingli Pan, Hui Wu, Shibing Wang, Xiangmin Tong, Shenghao Wu

**Affiliations:** 1 Department of Clinical Laboratory, School of Medicine, Affiliated Hangzhou First People’s Hospital, Westlake University, Hangzhou, 310014 China; 2 Zhejiang Provincial People’s Hospital (Affiliated People’s Hospital), Hangzhou Medical College, Hangzhou, 310014 China; 3 Postgraduate Training Base Alliance of Wenzhou Medical University, Wenzhou, 325035 China; 4 School of Basic Medical Sciences and Forensic Medicine, Hangzhou Medical College, Hangzhou, 310014 China; 5 Department of Hematology, The Dingli Clinical College of Wenzhou Medical University, The Second Affiliated Hospital of Shanghai University, Wenzhou Central Hospital, Wenzhou, 325000 China

**Keywords:** Hyaluronidase, Hyaluronic acid, Tumor microenvironment, Extracellular matrix, Tumor immunotherapy

## Abstract

Hyaluronidase (HAase), a family of enzymes critical for regulating physiological and pathological states, catalyzes the degradation of hyaluronic acid (HA), a key component of the extracellular matrix (ECM). By modulating ECM composition and cellular signaling pathways, HAase plays a pivotal role in diverse biological processes, including wound healing, tissue regeneration, and tumor progression. This review systematically elucidates the classification, biological sources, structural diversity, and catalytic mechanisms of HAase, emphasizing its dynamic involvement in disease pathogenesis and diagnostic potential. Furthermore, the article explores innovative therapeutic strategies centered on HAase modulation. HAase inhibitors emerge as promising tools for maintaining HA homeostasis, with implications in anti-inflammatory, antimicrobial, and antitumor therapies by blocking excessive HA degradation. Concurrently, HAase-mediated drug delivery systems represent a paradigm shift in overcoming biological barriers, enhancing bioavailability, and optimizing therapeutic outcomes through ECM remodeling. Notably, the synergy between HAase and immunotherapeutic modalities, such as checkpoint inhibitors and adoptive cell therapies, demonstrates synergistic antitumor effects by reshaping the tumor microenvironment (TME) and augmenting immune cell infiltration. Nevertheless, numerous challenges persist in the clinical application of hyaluronidase, including its immunogenicity, safety, application limitations and ethical considerations. This review synthesizes current research advances and unresolved issues, integrating molecular insights with translational perspectives, aiming to provide a more comprehensive and in-depth understanding of hyaluronidase and to advance clinical therapeutic strategies for hyaluronidase.

## Introduction

Hyaluronic acid (HA) is a hydrophilic glycosaminoglycan, composed of unbranched repeating units of D-N-acetylglucosamine and D-glucuronic acid. As a major component of the extracellular matrix (ECM), it participates in fundamental biological processes including wound healing, tissue regeneration, and cell signaling [1–3]. Hyaluronidase (HAase) is a glycosidase that can degrade the glycosidic bonds in hyaluronic acid polymers.As the majority of hyaluronidases belong to glycoside hydrolase (GH) families, while a minority are classified within polysaccharide lyase (PL) families [4, 5], their classification within the CAZy database reflects evolutionary relationships and structural correlations at the molecular level [6]. Hyaluronidase is a key enzyme in the regulation of HA dynamic equilibrium [7]. In 1928, Duran-Reynals first discovered hyaluronidase in testicular extracts and bacterial filtrates, which was then referred to as the “diffusion factor” due to its role in promoting the diffusion of substances such as vaccines, dyes, and toxins [8]. In 1940, it was officially named hyaluronidase by Chain and Duthie [9].

As a matter of fact, hyaluronidase is abundant in nature and has been found from complex higher animals to lower microorganisms [10]. In recent years, the research on hyaluronidase has become a extensively studied, and its function and properties have been gradually revealed. HAase has applications in medical fields including drug permeation enhancement, cosmetic surgery, and tumor immunology [7]. This review comprehensively examines the structural diversity of hyaluronidase and its role in physiological and pathological processes, with an emphasis on the therapeutic application of hyaluronidase, in particular focusing on novel therapeutic strategies targeting hyaluronidase activity or the HA degradation pathway in tumor immunotherapy. At the same time, many challenges and thoughts on the clinical application of hyaluronidase are also discussed.

## Classification, biological sources, and structure of hyaluronidase

### Classification systems of hyaluronidase

There are many classification systems of hyaluronidase in the world, mainly including four mainstream methods according to the source of enzyme, optimal reaction pH, specificity and catalytic mode, and amino acid sequence homology. Origin-based classification categorizes these enzymes into three domains: eukaryotes, prokaryotes, and viruses [10]. PH-based classification can be divided into acid enzymes (pH 3.0–4.0, such as human liver, serum and some animal toxin sources) and neutral enzymes (pH 5.0–8.0, the majority) [11]. However, classification according to substrate specificity and catalytic mechanism holds fundamental significance for understanding functional divergence among hyaluronidases [12].

**Fig. 1:**
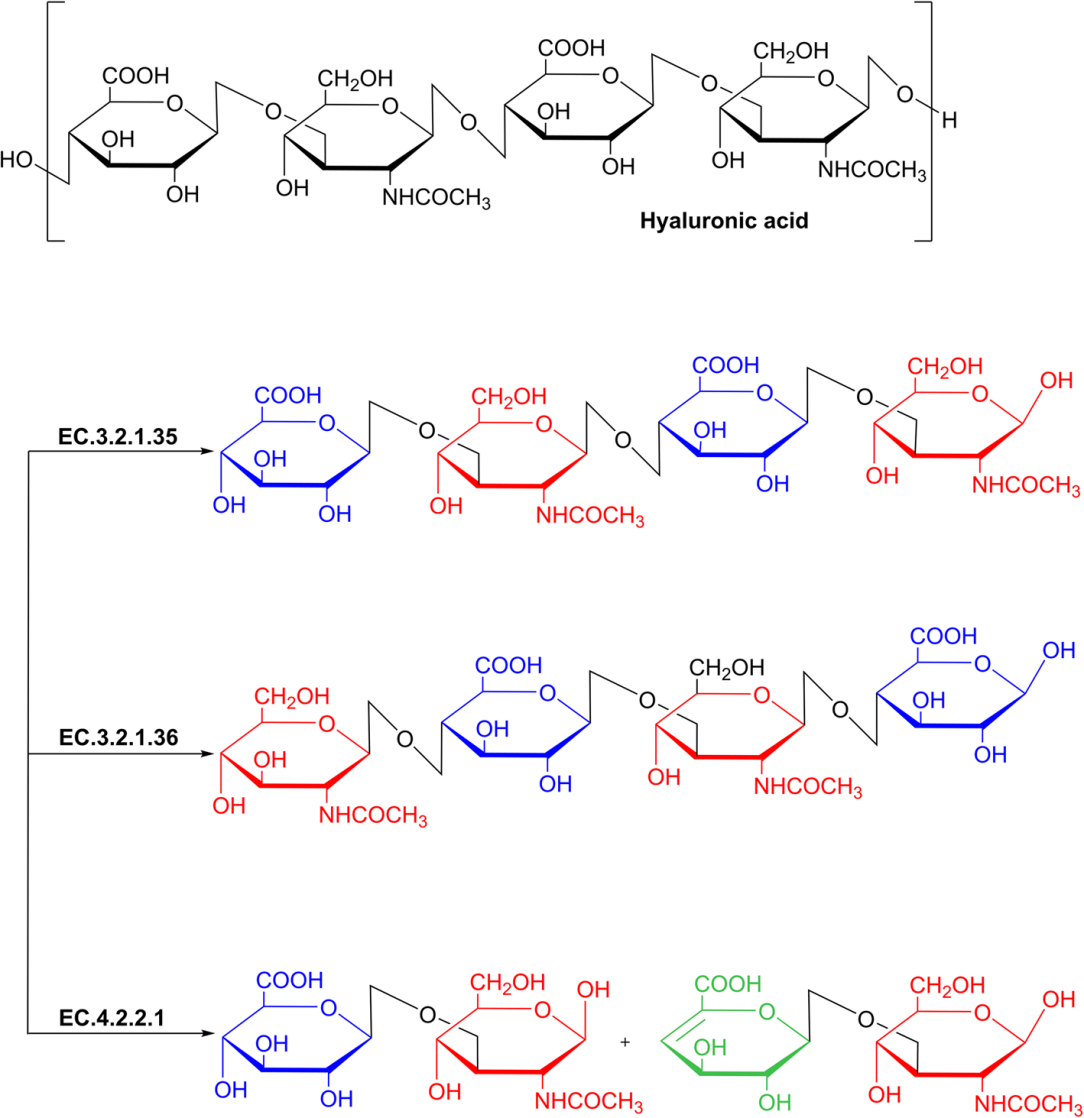
Schematic of the chemical structure of hyaluronic acid, and the classification of hyaluronidase based on enzyme specificity and catalytic mode

The most classical classification system, established by Meyer et al. in 1971 [12], categorizes hyaluronidases into three principal types based on substrate specificity toward HA and fundamental catalytic mechanisms (Fig. 1). Hyaluronoglucosaminidase (EC 3.2.1.35): A glycoside hydrolase (GH-class) predominantly sourced from mammals and venoms. It specifically hydrolyzes β−1,4-glycosidic bonds in HA, yielding even-numbered oligosaccharides (tetra-and hexa-saccharides) with N-acetylglucosamine as the reducing end [13, 14]. Hyaluronoglucosidase (EC 3.2.1.36): Represented by leech salivary enzymes [15], this GH-class hydrolase selectively hydrolyzes the β−1,3-glycosidic bond in HA, producing oligosaccharides with glucuronic acid as the reducing end, with hyaluronate tetra-saccharide as the major final product [16]. In stark contrast to the former two hydrolysis mechanisms, Hyaluronate lyase (EC 4.2.2.1): A polysaccharide lyase (PL-class) of microbial origin, which cleaves β−1,4-glycosidic bonds in HA through an elimination mechanism, generating non-reducing end products with unsaturated carbon–carbon bonds, as well as an unsaturated HA-disaccharide, namely 2-acetamido-2-deoxy-3-O-(β-D-gluco-4-enepyranosyluronic acid)-D-glucose [17, 18]. This catalytic dichotomy—hydrolysis versus β-elimination, coupled with bond-specificity (β−1,4 vs. β−1,3)—reveals fundamentally distinct degradation pathways and product chemistries. Notably, CAZy database-based classifications (GH/PL family) are highly associated with this functional classification, EC 3.2.1.35 maps to GH16/GH56/GH84 families; EC 3.2.1.36 exclusively resides in GH79 family; and EC 4.2.2.1 distributes across PL8/PL16/PL31 families [19–22].

### Common biological sources of hyaluronidase

#### Mammalian hyaluronidases

In the human genome, six different hyaluronidase genes have been identified, namely HYAL1, HYAL2, HYAL3, HYAL4, PH-20 (sperm adhesion molecule 1,SPAM1), and HYALP1(or HYAL6), with homology between 33 and 44% [4]. HYAL1 is widely present in plasma, urine, liver, kidneys, spleen, and heart. It is the main hyaluronidase that regulates the metabolism of hyaluronic acid in ECM. In addition, it is closely related to tumor biology, lysosomal storage diseases, and wound healing [23]. HYAL2 is primarily located in the liver, kidneys, spleen, and adult brain, where it is significantly expressed in lysosomes or on the cell membrane [24]. Moreover, HYAL2 is related to tumor biology and angiogenesis [23, 25]. PH-20 exhibits the strongest biological activity, existing in high concentrations in the testes, and is localized to the head and acrosome of human sperm, while there is no distribution in the midpiece and tail. PH-20 is a multifunctional membrane protein that not only degrades HA but also provides HA-induced signaling receptors [25, 26]. Compared to HYAL1, HYAL2 and PH-20, the biological activity and functional significance of HYAL3, HYAL4, and HYALP1 are relatively lower [27]. HYAL3 is primarily distributed in the testes and bone marrow, and is considered a non-enzymatic regulator of HYAL1, although its catalytic performance has not been clearly defined in current research [28]. HYAL4 is distributed in muscles and the placenta and is considered a chondroitinase, with no biological activity on hyaluronic acid [25]. HYAL6 is distributed in the testes and bone marrow, and due to the deletion of a segment of an exon during the human evolutionary process, the amino acid translation process is terminated prematurely and only transcribed but not translated, rendering it a pseudogene [29].

In addition to the six canonical hyaluronidase gene, KIAA1199 and transmembrane protein 2 (TMEM2) show similar roles to hyaluronidase in the degradation of HA [18]. KIAA1199, a deafness gene of unknown function, is mainly expressed by dermal fibroblasts in normal skin and has been shown to have a key role in the depolymerization of HA [30]. In 2017, the hyaluronidase activity of TMEM2 was first identified [31], and TMEM2 has been linked to the catabolism of HA [19]. Recently, a major controversy has arisen about whether TMEM2 is a hyaluronidase, Niu et al. arguing that TMEM2 clearly lacks hyaluronidase activity [32]; Sato et al. arguing that human TMEM2 is not a hyaluronidase but a regulator of HA metabolism [33]; Takuma et al. overturning the above conclusions, arguing that TMEM2 is a bona fide hyaluronidase enzyme and has intrinsic catalytic activity [34]. Therefore, whether human TMEM2 is a hyaluronidase and its catalytic activity need to be further studied.

Unlike humans, mice have seven genes that encode hyaluronidase. In addition to the six genes already identified in the human genome, the seventh hyaluronidase gene is HYAL5, which shares high homology similarity with other types of hyaluronidase genes [35]. HYAL5 is closely related to the success of fertilization in the house mouse, and spermatozoa with deletion of the PH-20 gene can be successfully fertilized as long as they are normally expressed with HYAL5. In addition to the house mouse, HYAL5 is also found in other rodents [36–38].

Currently, most of the marketed hyaluronidases are derived from mammalian testis, especially testicular extracts from bovine and sheep show high enzyme activity [39]. Bovine testicular hyaluronidase (BTH) has the widest clinical application, with sequence homology to human hyaluronidase ranging from 22.9% to 25.2% [40].

#### Venom hyaluronidases

The presence of hyaluronidase has been found in the venom of many animals, such as bees, spiders, snakes and lizards [41–43]. Most of the animal venom hyaluronidases not only degrade HA, but also other glycosaminoglycans such as chondroitin and chondroitin sulfate [44]. The full gene sequence of venom hyaluronidase has now been described, which has led to further structural understanding of venom hyaluronidase [45]. Venom hyaluronidase is an allergen that causes IgE-mediated severe or fatal allergic reactions in humans [46, 47]. Because of its role as a “diffusion factor”, hyaluronidase further exacerbates allergic reactions by facilitating the penetration of other harmful venom components and enhancing their entry into the bloodstream in various tissues [48]. Therefore, researchers have been working on the discovery of hyaluronidase inhibitors for use in antivenoms [49].

#### Leech hyaluronidases

Leech hyaluronidase (LHyal) was first discovered in leech extracts by Claude in 1937 [50]. The study of its enzymatic properties after isolation and purification revealed that LHyal has the advantages of high activity, high substrate specificity, no transglycosidase activity, and a more concentrated molecular weight distribution of its hydrolyzed products [51–53]. LHyal is sensitive to metal ions (Mn^2+^, Cu^2+^, and Fe^3+^), and has an optimal temperature of 45 °C and an optimal pH of 6.5 [52]. Currently, it has been hypothesized that leech hyaluronidase has acetylheparinase activity, but the ability to cleave heparin or acetylheparin sulfate has not been found [4]. Recombinant leech hyaluronidase is of great significance in clinical therapy due to its substrate specificity and it can eliminate the risk of cross-infection in animals [16].

#### Microbial hyaluronidases

Hyaluronidase is widely distributed in microorganisms, and microbial hyaluronidase (mHyal) is present in bacteria, fungi, and phages. Jiang et al. [18] characterized microbial HAases (mHyals) as Mn^2^⁺/Ni^2^⁺-sensitive enzymes with optimal activity at 37–45 °C and pH 5.5–7.0 [54], mHyals serve as virulence factors that mediate bacterial invasion, tissue penetration, and dissemination [55, 56]. mHyal can reduce the hardness and density of ECM by degrading HA, thus breaking through the physical barrier and even directly contacting cellular tissues [57]; it can also utilize polysaccharide utilization loci (PULs) as a nutrient source for survival [58], and create a suitable environment for its own growth and reproduction through the immune-inflammatory response [59–61]. Gut microbial mHyal can remodel intestinal microbiota by enriching short-chain fatty acid (SCFA)-producing bacteria (e.g., Bifidobacterium), suggesting therapeutic potential for bacterial colitis [62–66]. In addition, mHyal plays an important role in inhibiting breast cancer growth [67, 68] and enhancing the permeability and targeting of tumor drugs [69, 70].

### Structural features of hyaluronidase

In the previous section, we have already mentioned that the structure and catalytic mechanism of hyaluronidases are closely related to amino acid sequence homology, and the molecular structures of hyaluronidases from different species sources vary, but usually include catalytic domains and some auxiliary structural domains [71]. The overall three-dimensional folding, the key active site residues and the catalytic mechanism of the CAZy classification method are usually strictly conserved [22]. Therefore, this part is mainly based on CAZy, Protein Data Bank (PDB) database for the classification and elaboration of the structural relationships of different families of hyaluronidases, which are detailed in Table 1.

**Table 1:** The structures of hyaluronidases from different carbohydrate-active families

EC No	Family	Structure	PDB ID	Catalytic group	Catalytic mechanism
EC 3.2.1.35	GH16	β-jelly roll [72]	6XOF [73]	Glu/Glu	Retaining mechanism
	GH56	(β/α)7 [74]	2ATM	Glu/Asp	
	GH84	(β/α)8 [75]	8P0L [76]	Asp/Glu	
EC 3.2.1.36	GH79	(β/α)8 [77]	7EYO [78]	Glu/Glu	
EC 4.2.2.1	PL8	(α/α)6 toroid + anti- parallel β-sheet [79]	1C82 [80]	His/Tyr, Asn	cis-β-elimination reactions
	PL16	Triple strand β-helix	4UFQ [81]	Arg/Tyr, Gln	
	PL31	Parallel β-helix [82]	-	His/Tyr, Arg	

*PDB ID* RCSB protein data bank identity number, *Glu* Glutamic acid, *Asp* Aspartic acid, *His* Histidine, *Tyr* Tyrosine, *Asn* Asparagine, *Arg* Arginine, *Gln* Glutamin

The catalytic structural domain of hyaluronidase is the key region for its core enzymatic function, which is responsible for directly recognizing and binding glycosaminoglycan substrates such as hyaluronic acid and catalyzing glycosidic bonds therein. The key structure of the catalytic domain, the catalytic group and the catalytic mechanism are the differences between hyaluronidases.

The overall structures of HA hydrolases are similar, but the catalytic mechanisms differ. Structurally, there are fewer reports on HA hydrolases belonging to the GH16 family, and only two GH16 family HA hydrolases derived from Penicillium spp. have been identified, and it is hypothesized that their structures are similar to the β-jelly roll [83]. Most HA hydrolases belong to GH56, GH79 and GH84, whose overall structures are similar to the TIM barrel fold, with differences only in the number of β/α folded sheets [16, 84, 85]. In terms of catalytic mechanism, the catalytic mechanism of all families of HA hydrolases belongs to the retention mechanism, but the types are slightly different. The GH16 and GH79 family of hyaluronanases is the classical retention mechanism, and the GH56 and GH84 family of hyaluronanases is the substrate-assisted retention mechanism [40, 85, 86].

The structures of HA lyases were significantly different, but the catalytic mechanism was the same. The PL8 family hyaluronidase structure consists of a torus (α/α)6/6 helical barrel and an antiparallel β-sheet 2 domains, while the PL16 family hyaluronidase structure consists of a three-stranded β-helix,PL30 family hyaluronidase structure has not been reported, PL31 family hyaluronidase is mainly composed of Parallel β-helix [79–82]. All HA lyases are catalyzed by cis-β-elimination reactions [22, 87, 88].

## Mechanism of action

The core function of hyaluronidase depends on its specific recognition and catalytic degradation of HA substrate [89]. However, the non-catalytic functionalities of hyaluronidase warrant equal attention. The key to understanding these functions lies in elucidating their molecular mechanisms of action. Building upon the structural foundation delineated in Sect. ”Classification, biological sources, and structure of hyaluronidase”, this chapter systematically explores the enzymatic properties of hyaluronidase, the fine molecular processes of catalytic reactions, the regulatory mechanisms of its activity and the extracellular matrix (ECM)-modulating effects mediated by this enzyme. A comprehensive analysis of these aspects will provide critical insights into the significance of hyaluronidase beyond its conventional hydrolytic function.

### Degradation pathways of hyaluronic acid

Hyaluronic Acid (HA) is a linear glycosaminoglycan formed by repeated N-acetyl Glucosamine and glucuronic Acid disaccharide units linked by β−1,3 and β−1,4 glycosidic bonds [1], different molecular weight of HA determines the difference in its function [3, 90]. There are three main mechanisms of HA degradation in humans: (1) hyaluronidase-mediated enzymatic degradation, (2) reactive oxygen species-mediated oxidative damage, and (3) systemic clearance in the liver and kidney [4].

#### Enzymatic degradation

**Fig. 2:**
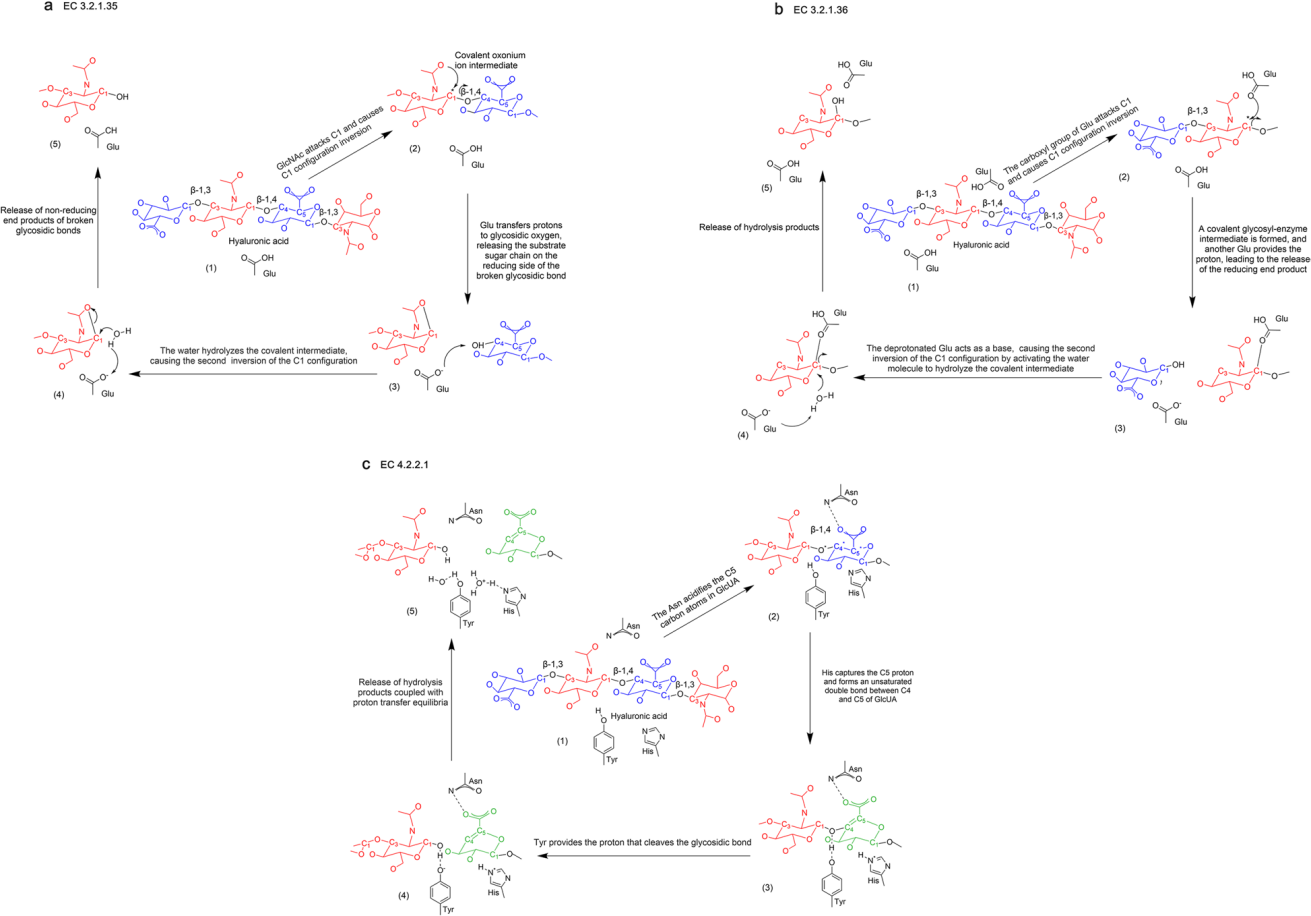
Molecular mechanisms of hyaluronic acid degradation by three distinct classes of hyaluronidases. a. EC 3.2.1.35 hydrolyzes β−1,4-glycosidic bonds via a retention mechanism; b. EC 3.2.1.36 hydrolyzes β−1,3-glycosidic bonds via a retention mechanism;c. EC 4.2.2.1 cleaves β−1,4-glycosidic bonds through β-elimination

EC 3.2.1.35 hydrolysis of β−1,4 glycosidic bond by retention mechanism, the β-configuration of the hydrolysate is consistent with the substrate [91], most of the reported animals and their venom-derived hyaluronidase belong to the GH56 family. The GH56 family HAases have many elongated clefts, known as binding clefts, which contain many positively charged hydrophobic amino acids [92]. Conserved in the binding cleft, the Glu catalytic residue acts as the proton donor, while the carbonyl oxygen atom of the acetylamino group in the substrate acts as the nucleophilic residue/base [93]. The specific steps are as follows (Fig. 2a): (1) HAase binds to the HA substrate. (2) Under the action of catalytic residues, the N-acetylglucosamine (GlcNAc) carbonyl oxygen of the substrate itself acts as an intramolecular nucleophile, attacking the heterologous carbon (C1) of the same sugar ring, forming a covalent oxonium ion intermediate accompanied by C1 configuration inversion, and completing the cleavage of the non-reducing side glycosidic bond of the glycosidic oxygen atom. (3) The protonated catalytic residue Glu transfers its proton to the glycosidic oxygen, releasing the substrate sugar chain on the reduced side of the broken glycosidic bond. (4) The water molecules in the active site hydrolyze the covalent intermediate, restore the catalytic residual matrix micronization state, prepare for the next round of catalysis, and trigger the second inversion of the C1 configuration. (5) The non-reducing end products of the broken glycosidic bond dissociate from the binding crack [6, 94–96].

EC 3.2.1.36 hydrolyzes the β−1, 3 glycosidic bond by the retention mechanism, and the heterocarbon configuration of its hydrolysis product is still retained. The catalytic process of the GH79 family hyaluronidase depends on the synergistic effect of two spatially separated conused Glu residues in the cleavage of the active site (Fig. 2b) [78]: One of them acts as a Nucleophile to directly attack the substrate hetero-carbon, forming a covalent glycosyl-enzyme intermediate; Meanwhile, another residue acts as an acidic carboxylate to provide a protonic group. In the subsequent hydrolysis step, the deprotonated acidic carboxylate acts as a base, launching a nucleophilic attack on the covalent intermediate by activating water molecules, triggering a secondary configuration inversion, and obtaining the hydrolysis product. This stepwise double substitution mechanism (nucleophilic substitution—hydrolysis) ultimately achieves the net retention of the heterocarbon configuration, and its essence is the superimposed effect of a two-step single configuration inversion [96–99].

EC 4.2.2.1 cleaves the β−1,4-glycosidic bond using a β-elimination reaction, forming an unsaturated double bond between the C 4 and C5 atoms at the non-reducing end of the product [100]. The binding cleft of the PL8 family is located at the junction of these two domains, the N-terminal and c-terminal, where there are many positively charged amino acids that can bind to negatively charged HA substrates, and the binding cleft is located at the junction of the N-terminal and C-terminal domains, and through the dynamic inter-domain movement to open and close the substrate binding crack, and finally cleave hyaluronic acid [101, 102]. But despite extensive research, the specific catalytic mechanism is not yet fully understood [5, 11, 80]. It has been hypothesized that the catalytic mechanism proceeds as follows (Fig. 2c): (1) HA forms a catalytic pre-reaction conformation through electrostatic complementary binding to the substrate-binding cleft. (2) The Asn acidifies the C5 carbon atoms in Glucuronic acid (GlcUA) near the catalytic site, triggering an electronic rearrangement. (3) The key His residue at the active site captures the GlcUA C5 proton, this proton transfer drives the formation of unsaturated double bonds between the C4-C5 of GlcUA, generating the characteristic 4,5-unsaturated oligosaccharide products. (4) During the synchronous cooperative proton transfer process, the tyrosine residues donate protons to the oxygen atoms of the glycosidic bond, resulting in the cleavage of the β−1,4-glycosidic bond. (5) The hydrolysis product dissociates from the active cleft, and balances hydrogen ions with the aqueous environment [101, 103].

#### Free radical depolymerization

The degradation of hyaluronic acid can also be achieved by free radical-mediated depolymerization, including a series of free radicals and oxidative reactive species (ROS), such as carbonate radical, hydroxyl radical, dichloride radical anion, dibromide and peroxynitrite [104–106]. HA chains undergo oxidative cleavage, a process that involves hydroxyl radicals attacking the C-H bond of the sugar ring, triggering β-glycosidic bond cleavage and producing polydisperse oligosaccharide fragments. In addition, high levels of ROS can induce hyaluronidase conformational changes or up-regulated expression in the inflammatory or tissue injury microenvironment, thereby inducing the enzymatic action of hyaluronidase on HA [107].

#### Clearance and metabolism of degradation products

Regardless of the degradation mechanism, the resulting HA fragments undergo efficient clearance. The generated HA fragments face two metabolic fates in the tissue microenvironment, 1. Local further decomposition: in the skin and other tissues, some small molecular fragments can continue to be degraded into monosaccharides; 2. Systemic lymphatic clearance: the vast majority of HA enters the lymphatic capillaries via interstitial fluid and is transported with the lymph to regional lymph nodes, where it is internalized and catabolized by lymphatic endothelial cells. Residual HA fragments enter the systemic circulation and are eventually degraded by liver endothelial cells. Multilevel scavenging systems ensure efficient metabolism of HA degradation products and maintain tissue homeostasis [108–113].

### Regulatory factors influencing activity

The activity of Hyaluronidase is regulated by many physiological, biochemical and environmental factors, mainly including the following aspects: (1) PH and temperature: HYAL 1–4 is an acid Hyaluronidase with the highest activity at pH 3–4, neutral hyaluronidases such as PH 20 and venom hyaluronidase are most active at PH 5–8 [25]. Most hyaluronidases generally exhibit optimal activity near a physiological temperature of 37 °C, with a rapid decline in activity above 45 °C due to protein deformation. However, hyaluronidase from some bacteria can tolerate high temperature, and even the optimum reaction temperature can reach 70 °C [54, 114, 115]. (2) Ions and cofactors: part of the hyaluronidase activity requires Ca2 +, Mg2 +, Zn2 + as cofactors, and the activity is dependent on NaCl concentration [116, 117]. (3) Redox status: Dysregulation and excessive generation of ROS can induce oxidative stress, perturbing intracellular redox homeostasis and subsequently promoting the activation and upregulation of hyaluronidase expression [118]. Conversely, antioxidants such as N-acetylcysteine and glutathione demonstrate inhibitory effects on hyaluronidase activity [119]. (4) Endogenous regulators: The most critical endogenous regulatory factors are hyaluronidase inhibitors, notably members of the Inter-α-inhibitor (IαI) family [120]. These inhibitors exert their suppressive effects through specific binding to hyaluronidase, thereby blocking its interaction with the substrate hyaluronic acid and effectively attenuating enzymatic degradation. (5) Signaling pathways and inflammatory milieu: Hyaluronidase expression and activity are frequently modulated by cellular signaling pathways, particularly those associated with inflammatory responses, while its enzymatic activity reciprocally influences inflammatory progression. During the early phase of acute inflammation, pro-inflammatory cytokines such as TNF-α and IL-1β activate signaling cascades (e.g., NF-κB pathway), leading to upregulated expression of hyaluronidase isoforms including HYAL1, HYAL2, and HYAL3. This creates a self-perpetuating cycle characterized by “elevated enzyme activity → accelerated hyaluronic acid degradation → aggravated inflammatory response [121]. However, during the resolution phase of inflammation, anti-inflammatory cytokines like IL-10 counteract this process by suppressing hyaluronidase production [122].

### Non-enzymatic functions of hyaluronidase

Beyond its canonical hyaluronan-degrading enzymatic activity, hyaluronidase exhibits diverse non-enzymatic functions that critically participate in physiological processes including cellular signaling, migration, proliferation, inflammatory responses, and immunomodulation [18]. It is noteworthy that these multifaceted non-enzymatic roles do not function in isolation, their mechanistic foundation lies in the direct regulation of extracellular matrix (ECM) physicochemical properties and signaling molecule homeostasis [7], with functional realization being highly dependent on this specific microenvironment. The ECM constitutes a highly specialized three-dimensional polymeric network, with collagen, fibronectin, laminin, proteoglycans, and glycosaminoglycans (GAGs) representing its principal components. Notably, HA serves as the predominant constituent of GAGs [123, 124].

By interacting with specific ECM components or anchoring directly to ECM structures, hyaluronidase effectively mediates cell-ECM communication and modulates downstream signaling cascades. Of particular relevance, the ECM not only provides structural support and mechanical integrity through components like HA but also actively participates in regulating cellular processes such as growth, differentiation, and dynamic remodeling [125, 126]. This bidirectional regulatory capacity enables hyaluronidase to exert broad physiological effects through alterations in ECM physicochemical characteristics and the generation of bioactive molecules. Consequently, the ECM serves as an indispensable integrative platform—combining physical, chemical, and signaling dimensions—for the manifestation of hyaluronidase’s non-enzymatic functionalities.

HA is an important “space filler” in ECM, the high viscosity and hydration capacity of HMW-HA maintains the mechanical support and compression resistance of the tissues. After the degradation of HA by hyaluronidase, the mechanical properties of the ECM change, the viscosity and elasticity decrease, which leads to the structural changes and affects the supportive role of the tissues [127–129]. In addition, the structure of ECM becomes loose, which leads to an increase in porosity and improves the permeability of ECM [130].

**Fig. 3:**
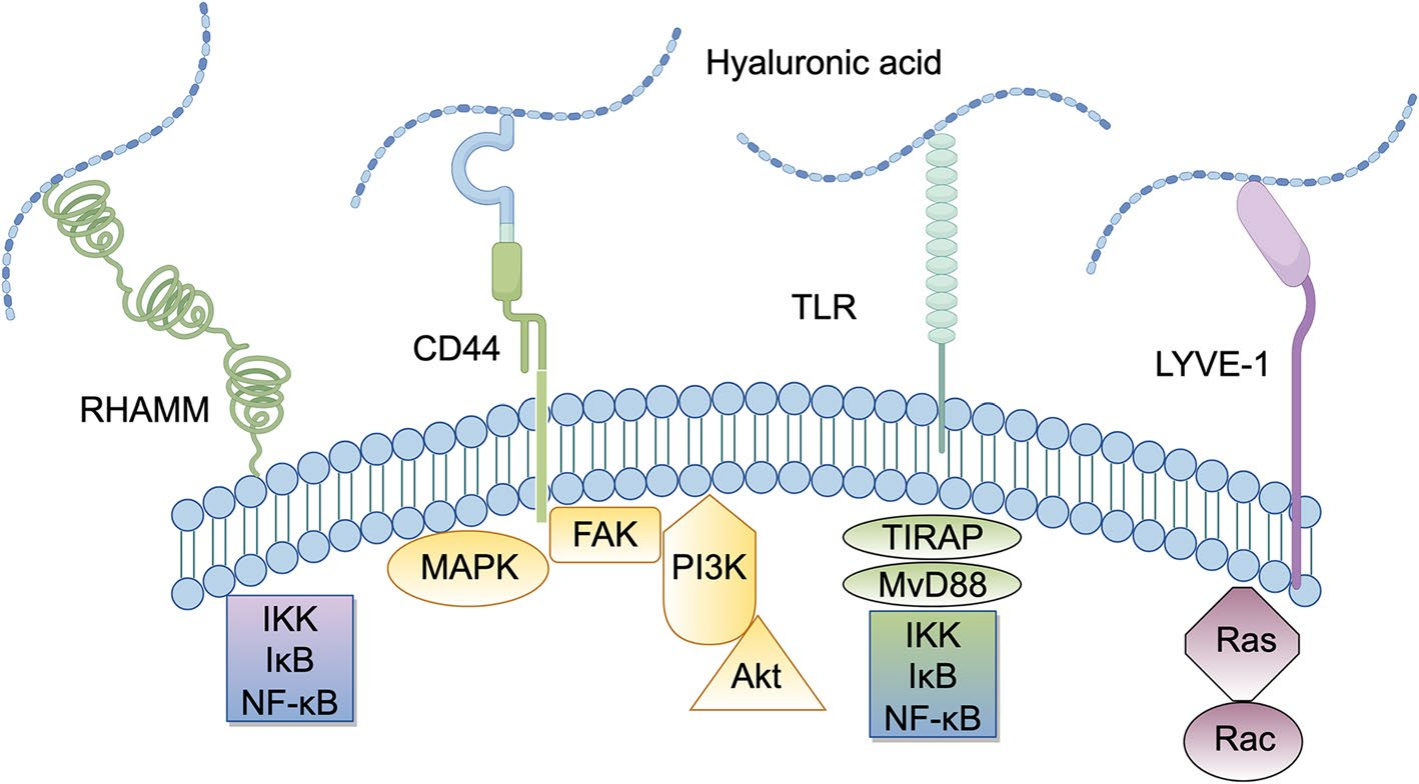
Interaction of hyaluronic acid and cellular receptor swaying.CD44 and RHAMM signaling pathways mediate the most complex and important cell-substrate interactions

Under normal physiological conditions, ECM has a dense physical barrier that limits cell migration, but when hyaluronidase acts on ECM, its loose structure reduces cell adhesion and promotes cell migration [131]. HA and its degradation products bind to a variety of cell surface receptor, such as CD44, RHAMM, TLR2, TLR4, LYVE,LAYN and STAB2 (Fig. 3) [132], thereby activating various signaling pathways and regulating cell–matrix interactions [133]. HMW-HA has antiproliferative and antivascular effects, whereas the degradation product, low molecular weight hyaluronic acid (LMW-HA), has a distinct role in promoting angiogenesis and cell proliferation [133–136]. Degradation products can promote the binding of Toll-like receptors (TLR2, TLR4) or CD44 that promote inflammation, activate signaling pathways such as NF-κb, and promote the release of inflammatory factors such as TNF-α, IL-1β, and IL-6 [137, 138]. The ECM also limits the entry of inflammatory cells under physiological conditions, and when ECM permeability is increased, the recruitment of inflammatory cells such as neutrophils and macrophages is enhanced, further exacerbating the inflammatory response [136, 139, 140].

## Role of hyaluronidases in disease

During physiological processes, hyaluronidase is involved in fertilization, adaptive immunity, platelet production, and remyelination [23]. Hyaluronidase plays a crucial role in the dynamic balance of HA. When the balance is disrupted, it will lead to a series of pathophysiological changes, which have an impact on the occurrence and development of the disease. In addition, hyaluronidase can also serve as a diagnostic marker for diseases and plays a significant role in the diagnosis of diseases.

### Malignant tumors

The global incidence of malignant tumors has witnessed a continuous surge in recent years, with approximately 20 million newly diagnosed cancer cases and 9.7 million cancer-related deaths reported worldwide in 2022 [141]. The effect of hyaluronidase on tumor cells can cause changes in the ECM, which in turn affects the composition and functional status of the tumor microenvironment (TME) cells [142]. Currently, the mechanisms of ECM resistance are the focus of research [143]. In Table 2, we elaborate on the components that contribute to ECM resistance and their role in resistance.

**Table 2:** The chemical components that lead to ECM resistance and their role in resistance

Chemical components	Mechanism of action	The impact on drug resistance
Hyaluronic acid	Forming physical barriers and regulate signaling pathways [144, 145]	Enhancing drug resistance and promote interstitial pressure [146]
Collagen	Regulating the integrin signaling pathway affects cell adhesion and migration [147]	Promoting the development of tumor stem cells leads to recurrence and resistance [148]
Fibronectin	Promoting resistance to nest loss apoptosis and activate pro-tumor signaling pathways [149–152]	Directly regulating cell adhesion-mediated resistance (CAM-DR) [153]
Laminins	Regulation of signaling pathways to alter cell adhesion, migration and differentiation [154]	Indirectly promoting resistance
Proteoglycans	Regulating the physical and chemical properties of ECM [155, 156]	Indirectly promoting resistance
Growth factors and cytokines	Regulating tumor growth, angiogenesis, and immune suppression [157, 158]	Enhancing the survival and proliferation ability of tumor cells

#### The double-edged sword of tumor-promoting and anti-tumor effects

**Fig. 4:**
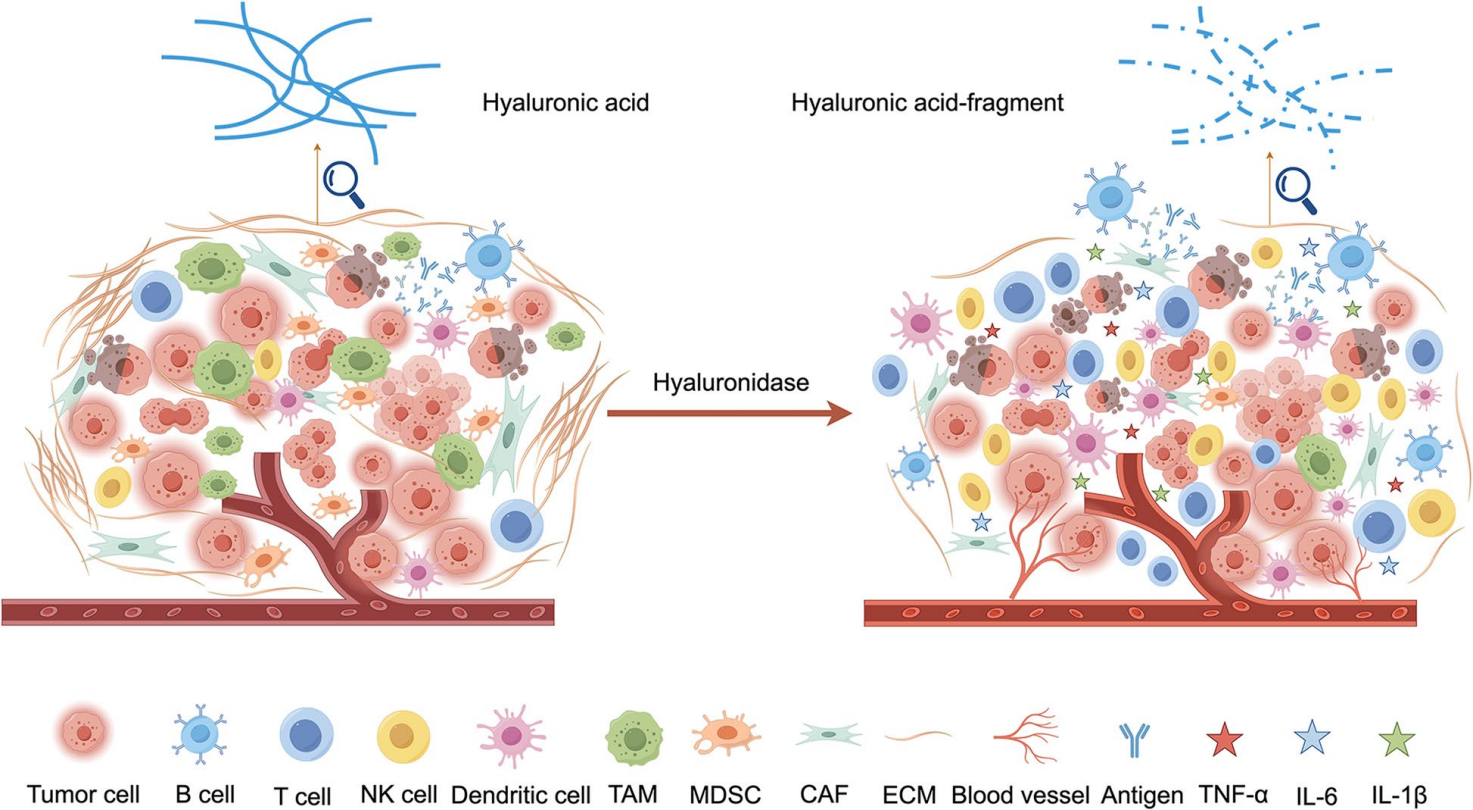
Effect of hyaluronidase-mediated degradation of hyaluronan on tumor microenvironment. HA degradation leads to ECM structural relaxation, alleviates the TME hypoxia state, and improves oxygenation and nutrient supply by promoting Neovascularization formation, which is beneficial to the development of the TME, it promotes the infiltration and activation of immune effector cells, including T cells, NK cells, DCs, and reduces the recruitment of MDSCs, which is also accompanied by the release of inflammatory factors such as TNF-α,IL-1β, and IL-6

In the TME, hyaluronidase-mediated degradation of HA regulates tumor progression and immune response through dual mechanisms. On the one hand, HA degradation leads to ECM structural relaxation, reduced tissue stiffness and interstitial pressure, and HA degraders bind to receptors on the surface of immune cells, such as CD44 and TLR2, which are important targets of HA degradation, activation of immune cell migration and activation of signaling pathways that promote infiltration and activation of immune effector cells, including T cells, NK cells, and DCs [159–162]. and reduced the recruitment of immunosuppressive myeloid-derived suppressor cells (MDSCs). At the same time, Tumor-associated macrophage (TAMs) undergo a shift from an M2 anti-inflammatory phenotype to an M1 pro-inflammatory phenotype, accompanied by the release of inflammatory factors such as TNF-α, IL-1β, and IL-6. ECM remodelling also alleviates TME hypoxia, improves oxygenation and nutrient supply by promoting Neovascularization, and enhances immune cell accessibility [163, 164] (Fig. 4).ECM loosening can increase the permeability of anticancer drugs and improve efficacy [165].

On the other hand, ECM degradation has pro-tumor effects, high ECM stiffness promotes tumor growth compared with soft ECM [166], and degradation products bind to CD44 or RHAMM, activation of Ras-MAPK, PI3K-Akt, FAK and other signaling pathways to enhance tumor cell motility [165, 167], it can also activate TGF-β, and it can also activate TGF-β, NF-κb and other signaling to induce epithelial-mesenchymal transition (EMT) of tumor cells, to make them more invasive and migratory [168]. New Blood vessels also enhance the supply of oxygen and nutrients to tumor cells and provide conditions for distant metastasis [169, 170].

#### Hyaluronidase as a biomarker for tumor diagnosis and prognosis

Among emerging biomarkers, hyaluronidases have garnered significant attention as research hotspots in tumor diagnosis due to their pivotal roles in shaping the TME. As a non-invasive diagnostic modality, combined urinary HAase activity and HA detection achieve 91% sensitivity and 83% specificity, outperforming conventional cytological examinations while enabling recurrence risk prediction [171, 172]. Elevated urinary HYAL1 levels in high-grade bladder cancer patients exhibit a 3–sevenfold increase compared to low-grade cases and healthy controls [173]. Recent studies further elucidate a dual functional role for HYAL3 in bladder cancer pathogenesis: its expression correlates significantly with immune cell infiltration status in the TME and serves as an independent prognostic factor for overall survival (OS) following multivariate analysis [174]. In prostate cancer, urinary HYAL1 emerges as a novel early diagnostic biomarker with independent predictive value [175], where elevated levels directly associate with increased disease probability and independently predict progression/recurrence risks [113, 176].

In breast cancer, augmented enzymatic activity of HYAL1 and HYAL2 correlates with aberrant signaling through HA receptors (CD44, RHAMM), thereby promoting tumor progression, metastasis, and recurrence. Notably, KIAA1199 (CEMIP) has been increasingly implicated in malignant progression across multiple carcinomas, including breast cancer, where its overexpression mediates invasive phenotypes [177–181]. TMEM2, transcriptionally activated by SOX4 in breast cancer, functions as a mediator of migratory and invasive processes, suggesting diagnostic potential for CEMIP and TMEM2 quantification [182].

For head and neck squamous cell carcinoma (HNSCC) [183], HYAL1 demonstrates potential as a tissue-specific biomarker, with elevated HYAL1 and PH20 expression observed in laryngeal carcinoma specimens [184]. In pancreatic cancer, hypomethylation of HYAL2 in peripheral blood constitutes a valuable early diagnostic indicator [185]. Non-small cell lung cancer (NSCLC) studies confirm KIAA1199 overexpression in tumor tissues correlates with poor differentiation, lymph node metastasis, and advanced staging, establishing its role as an independent prognostic predictor [186].

Similarly, gastric cancer exhibits significant upregulation of KIAA1199 expression, which has been validated as a key independent predictor of clinical outcomes. Mechanistically, KIAA1199 activates the canonical Wnt/β-catenin signaling axis, upregulating matrix metalloproteinase (MMP) family proteolytic activity to induce epithelial-mesenchymal transition (EMT) phenotypic conversion—a process strongly associated with enhanced metastatic potential [187].

### Infectious diseases

Hyaluronidase is an important “diffusion factor” for pathogens to invade the host [57]. As early as 1950, some researchers suggested that hyaluronidase could enhance the invasiveness and virulence of hemolytic streptococci [188]. Coleman et al. recently showed in the Macaca Nemestrina model that Group B streptococci (GBS), which has high expression of hyaluronidase, is more invasive during pregnancy and ultimately causes preterm birth [189]. Hyaluronidase also causes bacterial invasion in Streptococcus pneumoniae [88]. Hyaluronidase in Staphylococci is also an important causative agent of respiratory tract infections [190, 191].

### Autoimmune and inflammatory diseases

Hyaluronidase also has a unique pathological role in autoimmune and inflammatory diseases. In patients with rheumatoid arthritis, HA and hyaluronidase activity and expression levels are significantly elevated [192, 193]. In scleroderma, patients have normal activity levels of HYAL1 in the early stages, but HYAL1 activity decreases in the late stages [194]. HYA1 deficiency has a beneficial role in the early stages of type 1 diabetes, which may be mediated by a variety of protective effects against early diabetes-induced damage to endothelial cells and the glycocalyx [195]. Petrey et al. found that mice deficient in HYAL2 were more susceptible to inflammatory bowel disease (IBD) and that HYAL2 could modulate the early inflammatory response in colitis by limiting leukocyte extravasation [196]. Serum hyaluronidase activity is also significantly elevated in patients with psoriasis [197].

### Cardiovascular diseases

It is well known that NO reduces vascular stiffness, and in patients with coronary artery disease, the level of hyaluronidase rises in patients, and hyaluronidase is negatively correlated with NO production, a mechanism that is also closely related to the development of hypertension [198]. In patients with aortic disease, the ratio of collagen and Hyal to hyaluronidase is increased [199]. Researchers found that in HYAL2 KO mice, heart valve dilation, atrial dilation, cardiac hypertrophy, ECM deposition, and resulted in increased mortality [200]. In HYAL2 deficient mice, diastolic dysfunction and heart failure develop earlier [201]. In addition, HYAL2 deficiency in humans may be associated with a rare heart disease, Cor triatriatum sinistrum, but the mechanisms involved still need to be further investigated [202]. In an IL-10-induced myocardial infarction model in mice, the reduction of HYAL3 levels promotes cardiac wound healing by inhibiting the inflammatory response [203].

### Respiratory diseases

In the human airway epithelium, the expression and activity of HYAL1,2, and 3 are increased, as in allergic asthmatic responses [121]. In a rat model of radiation-induced lung injury, HYAL2 is involved in HA turnover during the early phase of lung injury and in the pathogenesis of pulmonary fibrosis [204]. Plasma HYAL1 levels are elevated in patients with Obstructive sleep apnoea (OSA) and further induce a systemic inflammatory response in OSA [205]. Elevated Hyal levels have been observed in patients with chronic obstructive pulmonary disease in both the stable and acute phases, and are particularly pronounced in the acute phase [206]. HYAL2 expression is elevated in both Idiopathic pulmonary fibrosis pulmonary fibrosis and pulmonary hypertension [207].

### Digestive System diseases

In addition to the previously discussed roles in IBD and cancers, alterations in hyaluronidase activity play a significant role in other digestive system disorders. Elevated levels of HYAL1 have been shown to promote autophagic responses in pancreatic acinar cells, exacerbating pancreatic secretion and inflammation, ultimately leading to pancreatic leakage in patients undergoing duodenectomy [208]. In the very early stages of toxic liver injury, hyaluronidase levels begin to rise on the first day and peak by the second day [209]. Compared to healthy individuals, patients with acute and chronic hepatitis C exhibit increased serum hyaluronidase levels during the early phases of the disease [171]. Furthermore, both HYAL1 and 2 levels are elevated in liver pathologies such as steatosis, steatohepatitis, and cirrhosis [210].

### Other diseases

In neurodegenerative diseases, hyaluronidase is an inhibitor in Alzheimer’s disease that can reverse endolysosomal dysfunction and protein lesions, alleviate memory impairment, and prevent Alzheimer’s disease, bring new tools to the treatment of Alzheimer’s disease [211]. In skin disease, HYAL1 and 2 expression is reduced in keloid tissue compared with normal tissue [212]. The metabolic regulation of HYAL1 and 2 has also been linked to the effects of ultraviolet radiation (UVB) on the skin, with increased levels of HYAL1 and 2 in the skin after exposure to UVB [213].

## Therapeutic strategy and application of hyaluronidase

### Hyaluronidase targeting strategies

HAase exhibits multidimensional therapeutic potential through the regulation of HA degradation and ECM remodeling in disease treatment. Its therapeutic strategies can be categorized as follows: pathological process intervention via inhibitors and drug delivery optimization using enhancers. The following sections discuss target classification, innovations in tumor immunotherapy, and clinical applications in non-oncological diseases.

#### Inhibitors: restoring hyaluronic acid homeostasis

Hyaluronidase inhibitors maintain HA homeostasis—particularly the dynamic equilibrium between HMW-HA and LMW-HA—by blocking excessive HA degradation. These inhibitors play pivotal roles in anti-inflammatory, antimicrobial, antitumor, and contraceptive drug development [214]. Current research has identified diverse structurally heterogeneous compounds with HAase-inhibitory activity, encompassing proteinaceous inhibitors, glycosaminoglycan derivatives, polysaccharides, botanical-derived bioactives, and synthetic organic compounds [95].

As early as 1946, Haas et al. first reported a heat-labile glycoprotein HAase-circulating complex in human plasma [215]. Later identified in mammals including mice, these complex exhibits magnesium dependency and protease sensitivity. Elevated activity levels of this complex correlate with hepatopathy and malignant neoplasms [216]. Heparin, a glycosaminoglycan, non-competitively inhibits HAase through interactions with surface amino acid residues rather than catalytic sites, with inhibitory efficacy diminished by HAase surface amino modification [217, 218]. O-sulfated HA fragments represent another documented inhibitor class exhibiting dose-dependent, mixed-type inhibition with potent activity profiles [219]. Certain anti-inflammatory agents (e.g., indomethacin, dexamethasone, sodium salicylate, and sulodexide) demonstrate HAase-inhibitory properties [11]. Vitamin C, an antioxidant, suppresses streptococcal and bovine testicular HAase activity [220].

Natural products constitute a critical reservoir of HAase inhibitors, offering structural diversity and favorable safety profiles. Alkaloids, terpenoids, and flavonoids dominate this category. Aristolochic acid, an alkaloid, non-competitively modulates snake venom HAase activity through mechanisms analogous to heparin, primarily engaging exposed tyrosine and tryptophan residues [221]. Inhibitory outcomes vary among terpenoids and flavonoids depending on HAase isoform specificity and concentration gradients [222]. Quercetin and apigenin exemplify clinically validated compounds with broad therapeutic applicability [223]. High-throughput screening by Jeremy et al. identified delphinidin, a natural anthocyanidin, as an HAase inhibitor that attenuates HMW-HA degradation, presenting novel antitumor opportunities [224]. Additionally, α-ketoglutarate derived from lactic acid bacteria exhibits anti-aging effects through HAase inhibition [225].

#### Enhancers: overcoming delivery barriers

The stratum corneum and ECM form dual barriers limiting drug penetration into deep tissues. HAase-mediated ECM remodeling represents a viable strategy to overcome these limitations. Currently, hyaluronidases used in clinical treatment include bovine-derived formulations, recombinant human hyaluronidase (rHuPH20), and PEGylated conjugates (PEGPH20) [226, 227]. The U.S. Food and Drug Administration (FDA) has approved nine HAase-containing drug formulations for enhanced delivery and large-volume injections [7].

Nanoparticles (NPs) are efficient local delivery systems owing to their nanoscale dimensions, biocompatibility, and surface modifiability [228]. These properties enable effective stratum corneum penetration, particularly for hydrophilic drugs and macromolecules [229]. Polymer-based nanocarriers, for example, encapsulate drugs to protect against environmental degradation while enabling controlled release [230, 231]. Synergistic strategies combining HAase with NPs include: 1) chemical conjugation of HAase to NPs for enzyme-carrier composite systems; 2) co-loading of HAase and drugs for enzymatic degradation-triggered release; and 3) pre-treatment of target sites with HAase to soften ECM and enhance NP penetration [232–234].

Nanocrystals (NCs), as submicron-sized carriers, significantly improve drug delivery by enhancing saturation solubility. Their application strengthens concentration gradients between skin and topical formulations, accelerating passive diffusion and prolonging tissue retention [235]. HAase-based liposomes (LPs) demonstrate clinical potential in intravenous delivery via two primary approaches: 1) HAase encapsulation on LP surfaces for composite systems, and 2) pre-injection of HAase at target sites for ECM remodeling [236, 237]. HAase-integrated NPs, NCs, and LPs represent promising strategies for drug delivery optimization. Beyond nanoscale systems, HAase research in non-nanoparticulate formulations—such as emulsions and microspheres—has advanced through matrix degradation kinetics, significantly improving transmembrane drug transport efficiency [238, 239].

### Innovative applications in tumor immunotherapy

The high morbidity and mortality rates associated with malignant tumors necessitate the exploration of novel therapeutic strategies. In recent decades, our understanding of tumors has been revolutionized, and we have discovered that tumors are complex ecosystems, the concept of tumors has shifted from a purely confined disease centered on tumor cells to a disease in which a variety of non-tumor cells interact with each other within the tumor [142]. Tumor immunotherapy, which targets the TME, has emerged as a highly effective therapeutic modality. Notably, the combinatorial use of HAase with immunotherapeutic agents has demonstrated synergistic antitumor effects by modulating ECM composition and enhancing immune cell infiltration.

In recent years, the focus of tumor immunotherapy has shifted from directly using drugs to kill tumor cells to combating tumors by achieving a lasting immune response in cancer patients, while also preventing metastasis and recurrence, making the treatment of malignant tumors enter a new era of precision medicine [240, 241]. Compared with traditional treatments such as surgery, radiotherapy and chemotherapy, tumor immunotherapy has the advantages of strong specificity and few side effects [242].

**Fig. 5:**
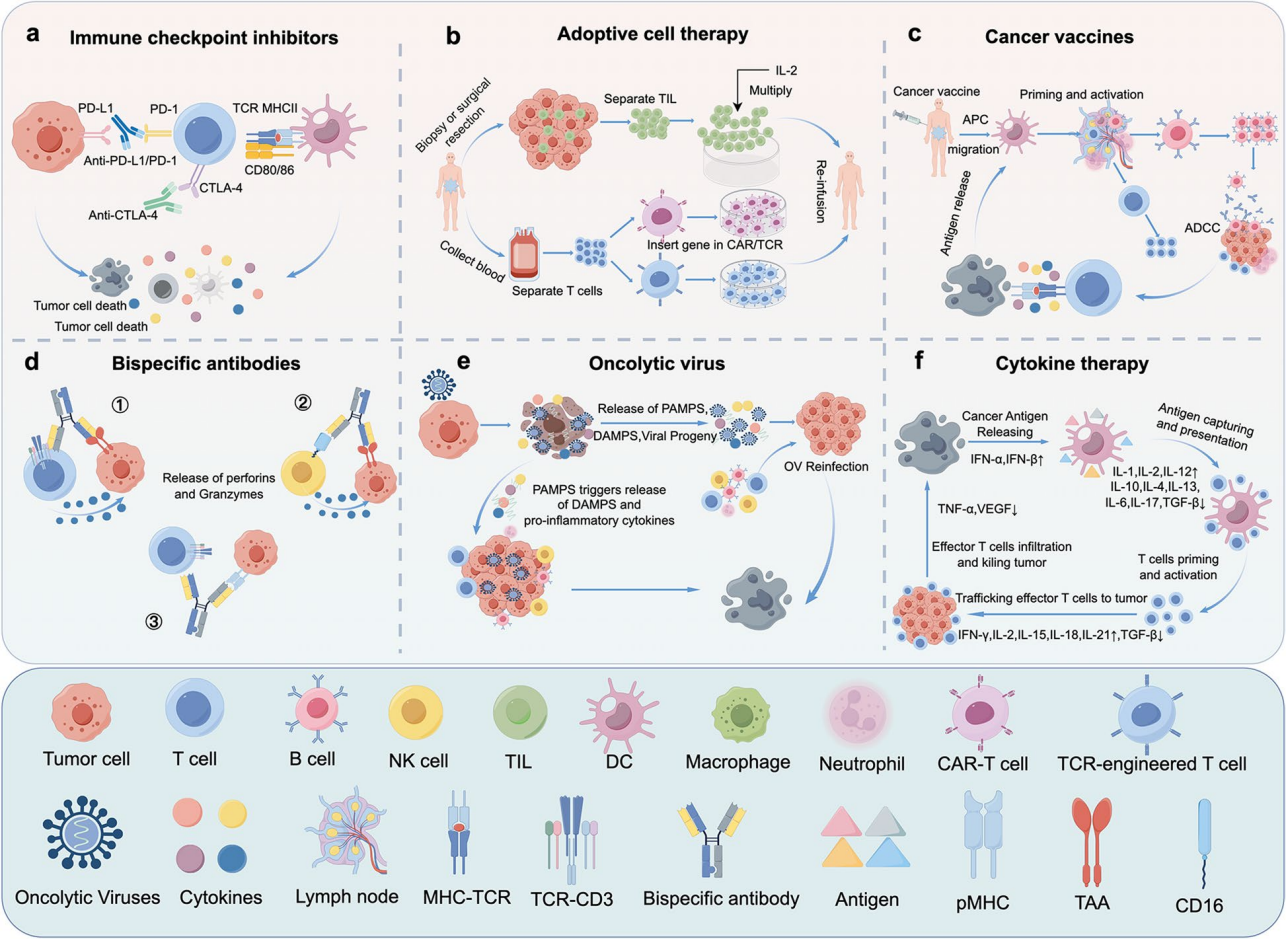
Mechanism diagrams of various tumor immunotherapy methods.a. Immune checkpoint inhibitor therapy reactivates the antitumor activity of T cells, thereby enhancing the Immune system’s attack on tumor cells; b. The patient’s immune cells are transformed, activated, and expanded in vitro and then re-infused into the patient to clear the tumor, including CAR-T and TCR-T therapies, among others; c: Mechanism of action of cancer vaccines; d. Different kinds of Bispecific antibodies; e. OVs are generally thought to mediate anti-tumor activity through two different mechanisms of action, one is selective replication within tumor cells, which has a direct lytic effect on n tumor cells; The second is to induce systemic anti-tumor immunity and anti-immune escape, including promoting The release of pathogen-related molecular patterns (PAMPs), danger-associated molecular pattern signals (DAMPs), and cytokines from tumor cells, thereby activating antigen-specific CD4 + and CD8 + T cell responses to regulate TME; f. Cytokine therapy works by activating immune cells through antigen capture and presentation

According to the different mechanisms of action (Fig. 5), tumor immunotherapy is divided into six main categories. (1) Immune checkpoint inhibitor therapy, by blocking immune checkpoint molecules, reactivating the anti-tumor activity of T cells, and thereby enhancing the immune system’s attack on tumor cells [243]. The U.S. Food and Drug Administration (FDA) approved immune checkpoint inhibitors include Cytotoxic T lymphocyte antigen 4 (CTLA-4), Programmed cell death 1 (PD-1), and its ligand Programmed cell death L1 (PD-L1) [244]. (2) Adoptive cell therapy (ACT), ACT is a form of passive tumor immunotherapy, in which immune cells originating from the patient’s tumor are transformed, activated, and expanded in vitro and then re-infused back into the patient’s body for the purpose of clearing the tumor [245–247]. (3) Cancer vaccines, cancer vaccines use tumor-associated autoantibodies (TAAS), tumor peptides or tumor cell lysis products to induce the body to produce tumor-specific immune responses, protect the body from tumor cell invasion, and achieve prevention and treatment of tumors [248]. (4) Oncolytic viruses (OVs), OVs can directly lyse tumor cells while retaining normal cells, and exert anti-tumor effects by releasing antigens and activating the tumor microenvironment [249]. (5) Bispecific antibodies: Bispecific antibodies can bind to two different antigens or epitopes at the same time. (6) Cytokine therapy: through the activation of natural immunity, enhancement of adaptive immunity, enhancement of antigen presentation, which are the three main mechanisms to play an anti-tumor role, including interleukins (ILs), interferons (IFNs), tumor necrosis factor (TNF) [250].

#### Augmenting immune checkpoint inhibitions

In preclinical studies, PEGPH20 in combination with PD-L1 inhibitors can significantly increase the sensitivity of breast cancer to PD-L1 inhibitors and significantly inhibit tumor growth, and reduced resistance to PD-L1 inhibitor monotherapy [251]. In triple-negative breast cancer, the simultaneous targeted delivery of hyaluronidase and TGF-β siRNA modified the tumor microenvironment, effectively enhanced the T-cell-mediated anti-tumor immune response and enhanced the efficacy of PD-L1 inhibitors when used in combination, and brought hope to triple-negative breast cancer patients, who have the highest metastasis and recurrence rates [252]. Hydrogel-encapsulated anti-CTLA-4 antibody has enhanced target delivery and anti-tumor effects in tumor-draining lymph nodes (TDLN) after the addition of hyaluronidase [253]. In locally advanced or metastatic non-small cell lung cancer, a phase III clinical trial of atezolizumab in combination with rHuPH20 for subcutaneous administration has been completed and demonstrated similar pharmacokinetic, efficacy, and safety profiles to those of intravenous atezolizumab, which opens up the possibility of improving the ease of treatment for patients. Excitingly, Tecentriq Hybreza™ (atezolizumab-hyaluronidase-tqjs) has been approved for marketing by the FDA in 2024 as the first subcutaneously administered PD-L1 inhibitor [254]. In advanced solid tumors such as metastatic melanoma, renal cell carcinoma, non-small cell lung cancer, hepatocellular carcinoma, colorectal cancer, and uroepithelial carcinoma, subcutaneous administration of nivolumab in combination with rHuPH20 has also yielded results that are not inferior to those of intravenous administration [255, 256]. In addition to in immune checkpoint inhibitors, subcutaneous formulations of hyaluronidase with other monoclonal agents such as trastuzumab-hyaluronidase-oysk [257], Darzalex Faspro (daratumumab-hyaluronidase-fihj) [258], Pertuzumab-Trastuzumab-Hyaluronidase-zzxf [259], rituximab and trastuzumab in combination with rHuPH20 [260] all showed similar efficacy and safety. rHuPH20 also increased the efficiency of absorption of cetuximab in subcutaneous administration [261].

#### Enhancing CAR-T cell infiltration

The combined use of hyaluronidase and adoptive cell therapy mainly focuses on CAR-T therapy. In existing preclinical studies, Xiong and colleagues found that the co-expression of IL-7 and PH20 in GPC3-targeted CAR-T (G3CAR-7 × 20) demonstrated significant anti-tumor effects in both in vivo and in vitro experiments, making it a potential treatment for hepatocellular carcinoma [262]. In addition, there are studies using bioorthogonal technology to couple α-PDL1 and HAase onto CAR-T cells, constructing H-P@CAR-T cells. Compared to traditional CAR-T cells, H-P@CAR-T cells exhibit better tumor infiltration ability, especially in mouse B-cell lymphoma (A20), where they enhance anti-tumor capabilities. In solid tumors, H-P@CAR-T cells have shown good therapeutic effects on mouse colon cancer [263].

#### Boosting cancer vaccine efficacy

Nanovaccines based on cancer vaccines are a hot research topic both domestically and internationally. Their smaller size makes them easier to be concentrated in lymph nodes, spleen and other lymphoid organs. Furthermore, dimensions comparable to pathogens promote efficient uptake by antigen-presenting cells (APCs), thereby improving the induction of anti-tumor immune responses [264]. The PEI/CpG/OVA (P/C/O) nanovaccine exemplifies a co-delivery system carrying both antigen and adjuvant. Within this construct, PEI enhances DCs internalization of the vaccine components. Concurrently, the CpG adjuvant is delivered to endosomes where it engages Toll-like receptor 9 (TLR9) [265, 266]. Critically, PEI mediates endosomal escape of the OVA antigen. This enables OVA processing and presentation via the major histocompatibility complex class I (MHC I) pathway, ultimately activating antigen-specific CD8 + T cells and amplifying the immune response [267]. By combining the PEI/CpG/OVA nano-vaccine with HAase, the infiltration of antigen-specific CD8 + T cells generated by the vaccine in tumor tissues has significantly enhanced the efficacy of in vivo anti-tumor immunotherapy. Hu et al. designed a new tumor nanovaccine and extracellular matrix scavenger. The main components of the nanovaccine are PEI/CaCO3/OVA/CpG (NVs), where PEI/CaCO3 nanoparticles not only efficiently deliver OVA and CpG to DCs, but also act as “autoluminescent” agonists to enhance the entire immune system [268]. PEG/PEI/pSpam1 (pSpam1@NPs), a new gene-mediated extracellular matrix scavenger, enables the isolation of polyethylene glycol by pH-responsiveness, which results in high expression of HAase in tumor tissues. Combining NVs with pSpam1@NPs promotes the infiltration of immune cells, especially CTLs, into tumors, and a stronger antitumor synergistic effect can be achieved [269]. It has been shown that by targeting the tumor extracellular matrix, hyaluronidase is able to activate tumor-draining lymph nodes (TDLNs), and the activated TDLNs can effectively enhance the immune response induced by tumor vaccines. When combined with a cryogel tumor vaccine, augmented systemic antigen-specific T-cell responses [270].

#### Amplifying oncolytic virus therapy

The combination of hyaluronidase and lysosomal viruses shows remarkable potential in tumor immunotherapy. Oncolytic adenoviruses armed with iRGD-modified hyaluronidase, have demonstrated strong tumor-specific CD8 + T cell activation and enhanced anti-tumor effects in immunocompetent mouse models [271]. The oncolytic adenovirus ICOVIR17 expressing hyaluronidase can also enhance the efficacy of oncolytic virus immunotherapy in refractory tumors such as glioblastoma by altering the extracellular matrix [272]. In other types of oncolytic adenoviruses, taking VCN-01 as an example, this oncolytic adenovirus, which combines hyaluronidase expression with RGD and HSG binding domain modifications, has shown significant anti-tumor effects across various types of cancer, such as pancreatic cancer, glioma, osteosarcoma, and melanoma, and has demonstrated good safety and efficacy in clinical studies [273–276]. It has also enhanced the effectiveness of chemotherapy using paclitaxel and gemcitabine [277]. In addition, the new generation of lysosomal adenovirus VCN-11 has hyaluronidase activity and is able to evade the attack of neutralizing antibodies, which further enhances the therapeutic potential of lysosomal viruses in the presence of neutralizing antibodies, and shows highly effective antitumor effects [278]. Previous studies have focused on lysogenic adenoviruses, and Wang et al. constructed a cowpox virus (OVV- HYAL1) encoding the hyaluronidase HYAL1, which promotes viral dissemination, chemotherapeutic drug diffusion, leukocyte infiltration, and immune activation in the TME. It showed promising anti-tumor effects and significantly prolonged survival in mouse models of pancreatic, breast and colorectal cancer [279].

#### Combination of hyaluronidase and other immunotherapeutic drugs

Although the clinical application of immune checkpoint inhibitors is in full swing, there are still many tumors for which immunotherapy still fails to achieve satisfactory therapeutic effects, the emergence of small molecules has made up for some of the shortcomings of immunotherapy [280]. Indoleamine 2,3-dioxygenase (IDO) is highly expressed in most human tumor tissues [281], and IDO activity is closely related to the regulation of cancer immune escape mechanisms and T-cell activity [282], therefore, IDO inhibitors have become small molecule drugs with therapeutic potential. Li et al. constructed a Sono-activatable semiconducting polymer nanoreshapers (SPNDNH) loaded with an IDO inhibitor (NLG919) and modified with hyaluronidase, which could be used as an anti-inflammatory agent for the treatment of cancer, promoting nanoparticle enrichment and immune cell infiltration by degrading abundant HA in the pancreatic cancer ECM, and also blocking IDO activity by NLG919, remodeling the tumor immune microenvironment, as well as the tumor immune microenvironment, tumor in situ growth and distant metastasis were suppressed in a mouse model of pancreatic cancer [283]. Induction of immunogenic cell death (ICD) is an effective method to activate TME, which can release antigens and DAMP, transforming “cold tumors” into “hot tumors” and enhancing immunotherapy [284]. Drugs capable of activating ICD such as doxorubicin (DOX), mitoxantrone, oxaliplatin, etc. have been extensively studied in tumor immunity [285]. A hyaluronidase-modified DOX-loaded silica nanocarriers (DOX@HMSPHs) have been designed, and the hyaluronidase on the surface of this nanomaterial promotes drug diffusion by degrading the HA in the ECM and decreasing the tumor mesenchymal pressure. Moreover, drug-loaded nanoparticles can release chemotherapeutic drug DOX continuously in acidic microenvironment, induce ICD of tumor cells, promote the maturation of DCs, and ultimately achieve anti-tumor therapeutic effect. In addition, the nanocarrier HMSPs can be used as an immune adjuvant to further promote the activation of tumor-infiltrating T lymphocyte in mice [286]. Dox@pssp-hh NPS is also a novel nano-delivery system that, in addition to promoting drug diffusion by reducing tumor stromal pressure, the highly expressed Glutathione within tumors can disrupt disulfide bonds in nanoparticles, thereby increasing drug delivery and drug resistance, promote structural changes in nanoparticles to release Dox, which is also associated with the production of reactive oxygen species, further inducing oxidative stress in tumor cells [287]. ZnO-DOX@HAase PEG-PAH-DMMA (ZDHD) NPs, also a nanocarrier that can deliver antitumor drugs deep into tumors and elicit ICD, was validated for antitumor effects in a mouse 4T1 tumor model. There was no histological damage to these organs, heart, liver, spleen, lungs and kidneys, after ZDHD treatment compared to the DOX alone treatment group, providing a new idea to improve the efficacy and reduce the adverse effects of chemoimmunotherapy for tumors [288]. Pts@DOXHANG@Gal is an engineered platelets (Pts)-based nano-aircraft, which is armed with Dox and the TGF-βri inhibitor GALUNISERTIB (Gal), redox-sensitive nanospheres (HANG) composed of hyaluronidase and bis-N-hydroxy succinimide are shed from the structure along with the GAL. Dox and Gal exerted antitumor effects through ICD and TGF-β inhibition and induced long-term immune memory [289]. Fluorouracil, folinic acid, irinotecan and oxaliplatin’s regimen (FOLFIRINOX) is an important treatment for metastatic pancreatic cancer, and a phase IB/II trial evaluated PEGPH20 in combination with FOLFIRINOX, surprisingly poor outcomes were seen, including greater toxicity and shorter median survival, so greater caution is needed when using hyaluronidase in combination with Oxaliplatin [290].

As of now, clinical trials combining bispecific antibodies with hyaluronidase are in full swing, particularly the star product Rybrevant (amivantamab) from Johnson & Johnson, which is being administered in a fixed subcutaneous combination with recombinant human hyaluronidase. These trials are currently recruiting patients with non-small cell lung cancer (NCT05498428). However, there have been no reports on the study of the combined use of cytokine therapy and hyaluronidase to enhance anti-tumor effects. In Table 3, based on data from ClinicalTrials.gov, we have listed representative clinical trial developments of hyaluronidase in tumor immunotherapy.

**Table 3:** Clinical trial progress of hyaluronidase in Tumor Immunotherapy

NCT	Combined treatment programmes	Cancer Type	Phase	Location
NCT03719131	Rituximab and hyaluronidase human (Rituxan Hycela)	Advanced Melanoma Undergoing Nivolumab and Ipilimumab Therapy	II	United States
NCT03656718	Nivolumab and rHuPH20	Non-Small Cell Lung Cancer(NSCLC), Renal Cell Carcinoma(RCC), Hepatocellular Carcinoma(HCC), unresectable or metastatic melanoma, MSI-H/dMMR CRC, melanoma	I/II	United States, Argentina, Brazil, Chile, France, Italy, Mexico, Netherlands, New Zealand, Poland, Spain, United, Kingdom
NCT03446040	Nivolumab and rHuPH20	Solid Cancers That Are Advanced or Have Spread [RCC, CRC, NSCLC, Triple Negative Breast Cancer (SCCHN), Squamous Cell Carcinoma of the Head and Neck (TNBC)]	I/II	United States, Australia, Canada, Japan
NCT06504394	Pembrolizumab and Hyaluronidase	Relapsed or Refractory Classical Hodgkin Lymphoma (rrcHL) or Relapsed or Refractory Primary Mediastinal Large B-cell Lymphoma (rrPMBCL)	II	United States
NCT06212752	Pembrolizumab and Hyaluronidase	NSCLC	III	Japan
NCT06099782	Pembrolizumab and Hyaluronidase	RCC, NSCLC, Melanoma	II	United States, Argentina, Australia, Chile, France, Japan, New Zealand, Poland, South Africa, Turkey
NCT05722015	Pembrolizumab and Hyaluronidase	Metastatic Non-small Cell Lung Cancer	III	United States, Argentina, Brazil, Chile, China, France, Guatemala, Hungary, Japan, Poland, Romania, South Africa , Spain, Thailand, Turkey
NCT04058964	Pembrolizumab and PEGPH20	Metastatic Pancreatic Cancer	II	United States
NCT03634332	Pembrolizumab and PEGPH20	Metastatic Pancreatic Cancer	II	United States
NCT02563548	Pembrolizumab and PEGPH20	NSCLC, Gastric Cancer	I	United States
NCT05340309	Atezolizumab and Recombinant Human Hyaluronidase	NSCLC	II	United States
NCT06099782	Atezolizumab and rHuPH20	NSCLC	II	Argentina, Brazil, Chile, China, Costa Rica, Greece, Guatemala, Hungary, Italy, Republic of Korea, Latvia, Mexico , New Zealand, Peru, Poland, Russian Federation, South Africa
NCT03267940	Atezolizumab and PEGPH20	Cholangiocarcinoma, Gallbladder Adenocarcinoma	I	United States, Republic of Korea, Thailand
NCT02045589	VCN-01(Genetically modified human adenovirus encoding human PH20 hyaluronidase) and Abraxane®/Gemcitabine	Pancreatic Adenocarcinoma	I	Spain
NCT02045602	VCN-01 and Abraxane®/ Gemcitabine	Locally Advanced Solid Tumors, Metastatic Solid Tumors	I	Spain
NCT03284268	VCN-01	Rrefractory Retinoblastoma	I	Spain
NCT03799744	VCN-01 and Durvalumab	TNBC	I	Spain
NCT05057715	VCN-01 and Human Chimeric Antigen Receptor Modified T Cells (huCART-meso)	Pancreatic Cancer, Serous Ovarian Cancer	I	United States
NCT05673811	VCN-01 and Nab-Paclitaxel, Gemcitabine	Metastatic Pancreatic Cancer	II	United States, Spain
NCT05498428	Amivantamab and rHuPH20	NSCLC	II	United States, Brazil, China, France, Germany, Israel, Italy, Japan, Republic of Korea, Malaysia, Spain, United Kingdom
NCT05388669	Recombinant Human Hyaluronidase and Amivantamab, Lazertinib	NSCLC	III	United States, Argentina, Australia, Brazil, Canada, China, France, Germany, Israel, Italy, Japan, Republic of Korea, Malaysia, Poland, Portugal, Spain, Taiwan, Thailand, United Kingdom,
NCT04606381	Amivantamab and rHuPH20	Advanced Solid Malignancie	I	United States, Canada, Republic of Korea, United Kingdom,

Current research findings indicate that the combined use of hyaluronidase with tumor immunotherapy in most clinical trials can enhance anti-tumor effects, increase patient survival, and improve prognosis. However, some clinical trials failed to show the superiority of hyaluronidase in enhancing the clinical activity of therapeutic antibodies, such as two MORPHEUS trials(NCT03193190 and NCT03281369). This discrepancy stems from three interconnected dimensions. (1) Trial design heterogeneity: insufficient sequential timing of key drugs. (2) Unstratified patient cohorts: Inclusion of HA-low tumors obscuring response in HA-high subsets. (3) Dynamic TME compensation: Rapid HA regeneration via HAS2 upregulation and collagen crosslinking [291–293]. Although the combination of atezolizumab and PEGPH20 failed to achieve the primary endpoint of improved objective response rate (ORR) compared to chemotherapy regimens in both studies, the signal-seeking trial design accelerated proof-of-concept evaluation while minimizing patient exposure to suboptimal therapies. The MORPHEUS platform further enabled randomized efficacy assessment of this novel combination within an adaptive framework [294].

### Clinical applications in non-oncological diseases

Hyaluronidase is widely used in medicine, enhancing the absorption and distribution of other medications. In addition to its applications in the field of oncology,it is valuable in ophthalmology, cosmetic surgery, anesthesia assistance [23]. Additionally, its low incidence of clinical adverse reactions has allowed hyaluronidase to enter the fast lane in clinical applications in recent years [18].

#### Enhance local infiltration anesthesia

The use of hyaluronidase to enhance surgical infiltration anesthesia is one of its most well-known effects. In 1951, Thorpe was the first to apply hyaluronidase in dermatological surgery [295], When hyaluronidase is used in combination with local anesthetic, the diffusion rate of local anesthetic is proportional to the amount of hyaluronidase injected, which accelerates the onset time of anesthesia and reduces the swelling caused by local infiltration [296]. This ensures the effectiveness and safety of local anesthesia, and now hyaluronidase has been widely used as an adjunct to local anesthetics, such as ophthalmology, dermatology, trauma surgery, and stomatology [23, 297].

#### Promote drug permeation and enhance drug bioavailability

Hyaluronidase acts as a “Diffusion factor” catalyzing the degradation of HA in the ECM, enhancing tissue permeability, thereby enhancing drug penetration and improving the bioavailability of injected drugs [130], it can also promote the dissipation of local accumulations of blood or fluid to eliminate hematoma and edema, and relieve pain at the injection site [298]. In addition to its use as a local anesthetic, hyaluronidase is also useful for the subcutaneous administration of drugs such as insulin, morphine, antibiotics, therapeutic monoclonal antibody, and immunoglobulins, accelerated circulation of drugs in synovial fluid speeds up pharmacokinetics, improves drug availability, and increases patient satisfaction with drug response [23, 299].

#### Application in the field of cosmetic plastic surgery

HA is a widely used soft tissue filler in the field of cosmetic surgery, applicable for facial remodeling, tissue enhancement, and deep skin hydration. However, improper operation can lead to side effects such as vascular occlusion, blindness, nodules, granulomas, delayed hypersensitivity reactions, and poor cosmetic results [300]. Alam et al. confirmed that hyaluronidase can remove nodules caused by hyaluronic acid [301]. When hyaluronic acid enters the ophthalmic artery, it is easy to cause embolism of the central retinal artery, and the injection of hyaluronidase from the back of the eye can degrade the hyaluronic acid in the blood vessel and surrounding tissues, so as to achieve the therapeutic effect [302]. Recently, it has been found that pretreatment with hyaluronidase at the recipient site of fat transplantation can improve the survival rate of fat transplantation [303].

## Challenges and considerations

### Potential side effects and safety concerns

In clinical application, the adverse reaction rate of hyaluronidase is low, and the most common side effect is anaphylaxis, but the incidence of anaphylaxis is only 0.05%−0.69% [304]. Hyaluronidase from different biological sources is one of the causes of allergic reactions [305]. Animal-derived hyaluronidase has the problems of low purity, high miscellaneous protein content, and strong immunogenicity, which can increase IgE-mediated immunogenic allergic reactions [23]. However, with the use of human recombinant hyaluronidase rHuPH20, the number of allergic reactions due to origin has been greatly reduced [306]. In allergic reactions, immediate hypersensitivity reactions are the most common, but delayed hypersensitivity reactions may also occur. The clinical manifestations of immediate hypersensitivity after hyaluronidase application include erythema, edema, urticaria, and in severe cases, can lead to anaphylaxis, treatment may include topical steroid creams or oral steroids or antihistamines [307]. Different injection sites and injection doses lead to different allergic reactions, and allergic reactions after ocular injections are the most reported, which may be related to the long residence time due to the dense blood vessels in the eye, and the main symptoms include edema, pain, and itching, etc., but there are also serious cases related to loss of vision, and even death, have been reported [308, 309]. When the local injection dose is less than 1500 IU, the allergic reaction is limited to the local area, but when high doses of hyaluronidase (200,000 IU) or intravenous injections are given, the risk of allergic reaction can rise to 31.3% [310]. Therefore, in order to avoid adverse reactions, an allergy skin test seems to be effective, especially in patients allergic to bee stings, where hyaluronidase is likely to be the allergen [311]. In addition, we should avoid injecting at the site of inflammation, adhere to strict hyaluronidase injection formulation and dosage control, and consider drug interactions, appropriate post-injection care can reduce the incidence of adverse reactions [312].

### Resistance and limitations in therapeutic applications

Animal-derived hyaluronidase can cause human immune response, lead to the production of neutralizing antibodies, affect the activity of hyaluronidase, and then cause allergic reactions and reduce drug permeability. In a related study of recombinant human hyaluronidase, treatment-induced rHuPH20 antibody responses could be observed, but no neutralizing antibodies were observed. It also has a low immunogenic potential in terms of CD4 + T cell activation and B cell cross-reactivity, so that long-term use is also well tolerated when rHuPH20 is used in combination with therapeutic agents [313, 314]. Because rHuPH20 has a short half-life and is easily degraded, repeated administration may be required, increasing the risk of local irritation and immunity, and reducing the risk of infection, the successful development of Polyethylene glycol recombinant human hyaluronidase PEGPH20 has extended the half-life of rHuPH20 from less than 3 min to 10.3 h, providing a new option for therapeutic modalities that require prolonged hyaluronidase maintenance [315]. Hyaluronidase may have the limitation of insufficient tissue selectivity for the unspecific recognition of normal and diseased tissues, especially in tumor immunotherapy, where combination with therapeutic modalities that can target tumor tissue is essential. In reversing hyaluronan complications, there are case reports of injections of high doses of hyaluronidase leading to total degradation of HA, which ultimately led to loss of therapeutic efficacy [316]. This also gives us the insight that multiple smaller doses of hyaluronidase can be used for treatment of non-urgent complications.

### Ethical considerations in medical use

There are many ethical issues to be concerned about in the medical use of hyaluronidase, and the safety risk and informed consent need to be considered first, the possible allergic reactions caused by animal-derived hyaluronidase, whether long-term application of hyaluronidase can degrade hyaluronan in normal tissues, such as skin laxity, decreased joint stability, etc. is not yet clear [309], patients should be fully informed of the risks and give informed consent before use. The ethical controversy surrounding animal-derived products can not be ignored. Bovine hyaluronidase may be involved in animal slaughter, may violate animal welfare, and may touch on certain religious and cultural taboos [317]. There have been no clinically confirmed cases, although cross-species infections with products of animal origin are thought to be possible and concerns have been raised about the carrying of unknown viruses such as prions [318]. So using rHuPH20 instead is a good choice. In addition, there are also ethical challenges with novel technologies, nanocarriers or gene-editing delivery systems may introduce unknown toxicities, and patients participating in early-phase clinical trials may bear high risks but limited benefits, which may increase the risk of adverse effects, strict Declaration of Helsinki is therefore required to ensure that trial designs are consistent with the risk–benefit ratio and that reversible interventions are prioritized [319].

## Conclusion

Hyaluronidase, a pivotal enzyme in the metabolism of HA, plays multifaceted roles in both physiological and pathological processes. Its structural diversity, derived from variations in catalytic domains and sequence homology across species, underpins its distinct mechanisms of action—ranging from hydrolytic cleavage to β-elimination. The dynamic degradation of HA by hyaluronidase profoundly impacts the ECM, including modulatiion of tissue permeability, immune cell infiltration, and cell–matrix interactions, which are critical in wound healing, inflammation, and tumor progression.

In oncology, hyaluronidase emerges as a double-edged sword that plays both pro- and anti-tumor roles, strategic exploitation of its ECM-remodeling ability offers promising therapeutic avenues. Innovations in tumor immunotherapy highlight hyaluronidase as a key adjuvant, enhancing drug permeability and immune cell infiltration. rHuPH20 and PEGPH20 have revolutionized subcutaneous drug delivery, improving the efficacy of immune checkpoint inhibitors and adoptive cell therapies. Furthermore, hyaluronidase-engineered oncolytic viruses and nanovaccines demonstrate synergistic anti-tumor effects by reshaping the immunosuppressive TME.

Despite these advances, challenges persist. Safety concerns, including allergenicity and off-target ECM degradation, necessitate rigorous dosing protocols and the development of species-specific recombinant enzymes. Resistance mechanisms, such as neutralizing antibodies against animal-derived hyaluronidases, underscore the importance of humanized formulations. Ethical considerations surrounding animal-derived products further advocate for synthetic alternatives. Future research should prioritize designing tumor-targeted hyaluronidase delivery systems to minimize systemic toxicity and enhance antitumor effects.

In conclusion, hyaluronidase stands at the nexus of ECM biology and translational medicine. Its dual role as a pathogenic driver and therapeutic ally underscores the need for precision in clinical applications. By harnessing its ECM-remodeling potential while mitigating adverse effects, hyaluronidase-based strategies may redefine standards in tumor immunotherapy.

## Data Availability

The results/data/figures in this manuscript have not been published elsewhere, nor are they under consideration by another publisher.
